# Benefit of the doubt: a new view of the role of the prefrontal cortex in executive functioning and decision making

**DOI:** 10.3389/fnins.2013.00086

**Published:** 2013-05-24

**Authors:** Erik Asp, Kenneth Manzel, Bryan Koestner, Natalie L. Denburg, Daniel Tranel

**Affiliations:** ^1^Department of Psychology, University of ChicagoChicago, IL, USA; ^2^Division of Behavioral Neurology and Cognitive Neuroscience, Department of Neurology, University of Iowa College of MedicineIowa City, IA, USA; ^3^Department of Psychology, University of IowaIowa City, IA, USA

**Keywords:** belief, doubt, executive function, heuristics, prefrontal cortex

## Abstract

The False Tagging Theory (FTT) is a neuroanatomical model of belief and doubt processes that proposes a single, unique function for the prefrontal cortex. Here, we review evidence pertaining to the FTT, the implications of the FTT regarding fractionation of the prefrontal cortex, and the potential benefits of the FTT for new neuroanatomical conceptualizations of executive functions. The FTT provides a parsimonious account that may help overcome theoretical problems with prefrontal cortex mediated executive control such as the homunculus critique. Control in the FTT is examined via the “heuristics and biases” psychological framework for human judgment. The evidence indicates that prefrontal cortex mediated doubting is at the core of executive functioning and may explain some biases of intuitive judgments.

## Introduction

Consider this statement, “Right now, there is a killer directly behind you.” Notice your automatic reactions upon understanding the sentence: your heart rate quickened, your pupils dilated, your hands became sweaty, and you may have even glanced behind yourself to make sure the statement was inaccurate (Kahneman, 2011). What is so remarkable about this chain of events is the implausibility of the accuracy of the statement. This author has no knowledge about the circumstances of its readers and the probability of the accuracy of the statement is vanishingly small. So why was your immediate, automatic reaction to consider the statement above as truthful? Although counterintuitive, recent social psychological research has revealed that the initial step of understanding something is inseparable from believing it (Gilbert, 1991; Gilbert et al., 1993), and only a secondary psychological act can produce disbelief or doubt to an idea. The act of understanding cognitions *is* the act of believing them. In this model, cognitions are active agents which will produce cognition-consistent behavior. A secondary psychological process can produce doubt toward these active cognitions which inhibit cognition-consistent behavior. The process of cognitive representation involves an initial belief, and if there are discrepancies between the initial belief and other mental representation, doubt can be retroactively affixed to this belief.

Under this psychological framework, the **False Tagging Theory** (FTT; Asp and Tranel, 2012) was developed as a neuroanatomical model of the belief and doubt processes. The FTT asserts that doubt is a secondary process governed by the prefrontal cortex via affective “false tags” (Damasio, 1994), which are affixed to cognitive and perceptual representations in association cortices in the parietal and temporal lobes (e.g., the temporal-parietal junction; TPJ). A key aspect of the model is that “**false tagging**” is a singular function that multiple modalities can access, use, and compete for (Asp and Tranel, 2012). “False tagging” is a limited resource which can be taxed during periods of high cognitive work. For instance, the prefrontal cortex is theorized to be critical for “false tagging” perceptual distractors to keep focused attention (Desimone, 1996; Coull, 1998) as well as “false tagging” inaccurate or disadvantageous cognitive information. When the difficulty to hold focused attention or make a choice during a decision-making scenario increases (e.g., by increasing the number of distracting representations or response option representations, respectively), more “false tagging” resource is consumed. If there is a concurrent requirement of both “false tagging” to perceptual and cognitive representations, there can be competition for the “false tagging” resource and the efficacy of each process may be decreased (e.g., Gilbert et al., 1993). The FTT aims to reconcile several functions frequently attributed to the prefrontal cortex with the singular resource of “false tagging”: inhibition, extinction learning, cognitive switching, memory retrieval monitoring, planning, decision-making, attentional focusing, and working memory maintenance (Asp and Tranel, 2012). The prefrontal cortex contributes to these psychological processes by “false tagging” or doubting automatically believed representations during the associative activations that are perpetually occurring in the mind (see the functions of dual-process models' **System 1**, Stanovich and West, 2000; Kahneman, 2011). The “false tagging” function is a specific component of the executive functions described above. The “common executive functioning” component described by Miyake and Friedman (2012), which has almost perfect overlap with inhibition processes in executive functioning tests, is theorized to be the “false tagging” function. However, overall, executive functions are (1) not specific to prefrontal cortex functioning (Collette et al., 2006), and (2) have additional components such as updating and set-shifting (Miyake and Friedman, 2012) that are independent of the inhibition or the “common executive functioning” component. The theorized prefrontal function of “false tagging” is only a specific part of the broader mechanics of an executive function (see below).

The FTT uses the psychological model of System 1 to posit three principles critical for the role of the prefrontal cortex in executive functioning: (1) *Principle of perpetual associative activations*; the mind is constantly activating representations in an associative manner toward mental representations of stimuli, relevant goals, other cognitions, and emotions. (2) *Principle of activated representations as beliefs*; the associative activations of representations are believed in the sense that they will produce cognitions, emotions, and behaviors that are consistent with each activation. (3) *Principle of regional segregation of function*; the activation of mental representations are conducted and stored outside of the prefrontal cortex (primarily in the association cortices of the parietal and temporal lobes). The prefrontal cortex works in concert with the parietal and temporal association cortices to doubt or inhibit cognitions but does not directly store semantic, episodic, and perceptual mental representations perpetually activated in System 1 itself. System 2, the rational and slower psychological system, is an umbrella concept for logical, rule-based, and abstract processes outside of System 1. The FTT posits that System 2 arises from the interaction of the prefrontal cortex's “false tagging” function and the associative activations in cortices outside of the prefrontal cortex. This framework allows for a common prefrontal cortex mediated “false tagging” function for various psychological functions (for further explanation of “false tagging” in psychological functioning, please see Asp and Tranel, 2012): (1) *Inhibition*; “prepotent” activations of System 1 must be “false tagged” to prevent unwanted action. (2) *Extinction learning*; activations of learned stimulus-outcome representations (that are no longer associated) must be “false tagged” to prevent continued action toward the learned representation. (3) *Cognitive switching*; activations of learned stimulus-outcome representations (that are no longer associated) must be “false tagged” to allow searching and learning of a new stimulus-outcome association. Here, the FTT predicts that the prefrontal cortex is critical for the inhibition of the old association rather than the ability to acquire a new one. (4) *Memory retrieval monitoring*; activations of mental representations during a memory search that are incorrect must be “false tagged” to prevent belief in the errant memory. This perspective assumes that memory searches associatively activate both subjectively correct and incorrect representations (Nadel and Moscovitch, 1997), which is consistent with System 1's associative but imperfect activation pattern. (5) *Planning*; activations of potential action representations that are either inappropriate for the context or out of temporal sequence must be “false tagged” to allow for appropriate, ideal actions for the context to be represented. (6) *Decision-making*; activations of disadvantageous decision-outcome representations must be “false tagged” to allow for advantageous, ideal decision-outcome representations to be selected. (7) *Attentional focusing*; activations of irrelevant stimuli representations must be “false tagged” to allow for continued focused attention to a particular mental representation. The FTT posits that the sole attentional function of the prefrontal cortex is to “false tag” (or inhibit) both cognitive and perceptual distractors. (8) *Working memory maintenance*; activations of irrelevant perceptual or cognitive representations during a delayed memory task must be “false tagged” to allow for continued maintenance of an item in working memory. The FTT suggests that working memory representations are not temporarily stored in the prefrontal cortex (Postle, 2006) but are stored in parietal and temporal association cortices (Ruchkin et al., 2003). The prefrontal cortex exclusively “false tags” or inhibits perceptual or cognitive representations are irrelevant to the critical memory representation and actively maintains working memory by representation filtration. A defining feature of the FTT is that prefrontal-mediated selection occurs exclusively via a negative bias toward inaccurate beliefs, disadvantageous response options, and irrelevant perceptions (for a positive bias model of selection, see Miller and Cohen, 2001). The prefrontal cortex solely works to eliminate representations that are not advantageous to the organism, which allows other representations to be acted upon.

Note that these executive functions are not necessarily mutually exclusive for optimal performance. Optimal cognitive switching involves the “false tagging” of an old stimulus-outcome association but it also may critically rely on “false tagging” irrelevant stimuli on the task or in the environment (to focus of task demands) or “false tagging” disadvantageous new alternative stimulus-outcome associations (in contexts with multiple options).

**Figure 1:**
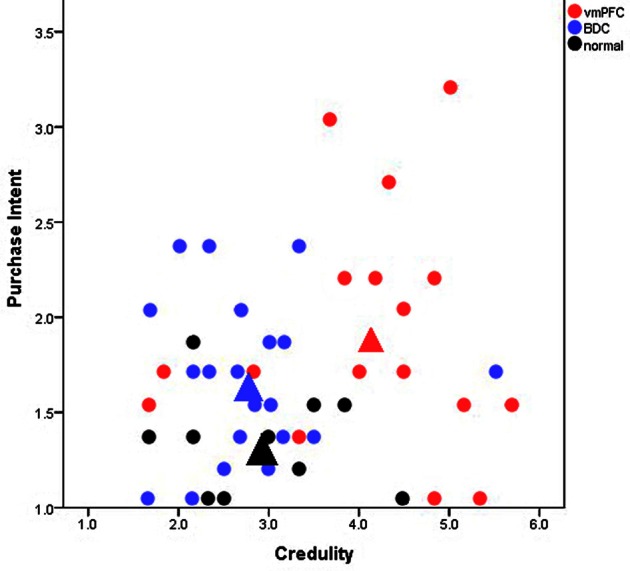
**Scatterplot of participants mean credulity and purchase intent toward misleadingly advertised products.** Each point represents an individual participant. Red, blue, and black circles represent patients with ventromedial prefrontal cortex (vmPFC) damage, brain-damaged comparison patients (BDC), and healthy comparison participants (normal), respectively. The large triangles represent the overall mean for each group. The *y*-axis extends from 1 to 5 with higher values corresponding to an increased intent to purchase the products in the ads and lower values to decreased purchase intention. The *x*-axis extends from 1 to 7 with higher values corresponding to increased belief in the ads (and more credulity) and lower values to increased skepticism. Patients with vmPFC damage where significantly more credulous to the misleading advertisements than comparison participants and displayed the highest intent to purchase the advertised products.

Our study, “A neuropsychological test of belief and doubt: damage to the ventromedial prefrontal cortex (vmPFC) increases credulity for misleading advertising” (Asp et al., 2012a), offers some of the first direct empirical evidence toward the FTT. In our view, the prefrontal cortex affixes “false tags” or doubt markers to cognitive representations; and, therefore, damage or dysfunction of the prefrontal cortex should produce decreased doubt that is accompanied by increased belief to novel external information. When given information that many people find dubious, such as farfetched claims on advertised products, individuals with damage to the prefrontal cortex (specifically the vmPFC), should fail to doubt the claims thereby creating increased credulity to them. To investigate this prediction, we gave patients with vmPFC damage, patients with damage to regions outside of the vmPFC, and healthy participants a series of magazine advertisements that were deemed misleading by the Federal Trade Commission. We found that vmPFC patients were more credulous and reported more purchase intention than the comparison groups even when the misleading ads contained a disclaimer rebutting the misleading claim (Figure 1). This result was not due to differences in demographic variables or general cognitive functioning, such as intelligence, memory, or reading ability. Rather the results were specific to the location of the lesions.

This study extends earlier evidence that prefrontal patients are vulnerable to believing get-rich-quick schemes (Damasio, 1994), religious dogma (Asp et al., 2012b), self-derived confabulatory statements (Gilboa and Moscovitch, 2002), and statements from individuals in positions of authority (Berlyne, 1972; Asp et al., 2012b). In addition, the FTT proposal that prefrontal patients have a **doubt deficit** recasts older research in new light. For instance, Stuss et al. (2001) found that prefrontal patients were markedly suggestible in response to an individual who was intentionally deceiving them. The researchers interpreted this result as a failure of theory of mind. Other investigations support this finding as prefrontal patients often are impaired when inferring others' thoughts, intentions, and feelings (Leopold et al., 2012). However, the FTT offers a potential alternative interpretation as susceptibility to intentional deception may be conceived as general doubt deficit regardless of whether there is an agent producing the deception (Asp et al., 2012a). Whether a failure to detect deception from a mindful agent is a product of a general failure to doubt or a failure to infer intentions (that are deceptive) is an important question that future research should address.

## Convergent evidence

### Evolutionary evidence

From a strictly logistics standpoint, a design of belief and doubt processes, where “all mental representations are beliefs” and doubt is retroactive, theoretically, is most adaptive to an organism (Gilbert, 1991). If belief is construed as two discrete processes of mental representation and positive assessment, where cognitions are first represented and then must undergo a separate assessment process before they are believed, the experience of representing, learning, and behaving would be extremely laborious. A positive assessment would need to be attached to virtually every representation that could be acted on or reacted to. Even perceptual representations would require an evaluation to enable the production of relevant behavior. Thus, this process would consume precious time and energy as one would need to assess whether the perceptual representation of a *bear running toward you* is in fact an actual bear running toward you. The perspective of “all mental representations are beliefs” avoids the evolutionary pitfalls of actually assessing everything that is represented.

In addition, the early evolutionary emergence of meaningful neural communication likely began by environmental antecedences directly producing reflex-type behavior without intermediate stages of assessment (Miller, 2009). Later development of inhibition to these early reflex circuits provided the first “executive” control and acted as proto-doubting devices in early neural circuitry (Hawkins et al., 2006). The FTT posits that these vestiges of early circuitry remain in human psychology as belief is primary and automatic whereas doubt is secondary and retroactive (Gilbert, 1991).

### Developmental evidence

The FTT suggests that “false tagging” or doubting (in the cognitive domain) is mediated by the prefrontal cortex. Individuals that undergo prefrontal cortex structural integrity or functionality declines or have underdeveloped prefrontal cortices should have a general doubt deficit which produces credulity to external information. First, life-span studies have shown that certain populations have an increased vulnerability to belief (Gilbert, 1991; Denburg et al., 2007). Children are often credulous, and skeptical thinking develops relatively late in childhood (Pea, 1980; Bruck and Ceci, 1999). Increased skepticism during early development parallels maturation in prefrontal cortex functioning (Diamond, 2002), as the prefrontal cortex is relatively underdeveloped early in childhood (Dempster, 1992; Giedd et al., 1999; Klingberg et al., 1999; Sowell et al., 1999). Certainly, credulity early in development is also attributable to a lack of basic knowledge, but it is just this inability to disbelieve without contradictory knowledge that supports the FTT and the view that initially understanding cognitions is the act of believing them. The FTT works on the principle of coherence (Gilbert, 1993), which states that disbelief results from the comparison of discrepant, mutually incompatible cognitions. A lack of incompatible cognitions in children leaves cognitions believed rather than simply represented without belief or disbelief. The influence of both knowledge and prefrontal cortex development may play complementary roles in the maturation of doubt, but for now, it is the province of future research.

Moreover, the process of early brain and psychological development would favor the “all mental representations are beliefs” perspective as it would be disadvantageous and perhaps disastrous if children could easily disbelieve basic knowledge of the world. If belief and disbelief were symmetrically opposed, good instruction such as *do not swim near the crocodiles* would be less likely to be believed and thus followed. Disbelieving representations without initial belief could dramatically slow cognitive development as knowledge and the relevant behavior from that knowledge must be learned at a rapid pace (e.g., Ganger and Brent, 2004). A design of “primitive” credulity followed by “acquired” skepticism (Bain, 1859) is the most adaptive developmental model for belief and doubt.

**Figure 2:**
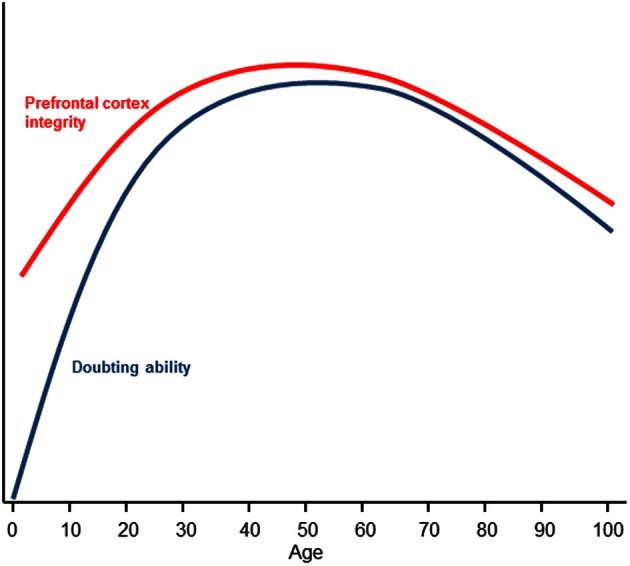
**Changes in doubting ability and prefrontal cortex integrity as a function of age.** Prefrontal cortex integrity is defined by white matter organization (Klingberg et al., 1999; Pfefferbaum et al., 2005) and is represented by the red inverted U. Doubting ability is represented by the blue inverted U. Early prefrontal integrity development is theorized to track doubting abilities but the initial absence of knowledge representations (which provide discrepant beliefs to form doubt) suggests a steeper ascending curve for doubting ability as knowledge is gained. Late prefrontal integrity development is theorized to be tightly coupled to doubting ability as knowledge is not appreciably diminished in older adults (e.g., Horn and Cattell, 1967).

At the other end of the lifespan, research has found that older adults are disproportionally credulous (Chen and Blanchard-Fields, 2000; Denburg et al., 2007). This finding has obvious and direct implications for older persons' vulnerability to financial fraud (Chen, 2007; Infogroup/ORC, 2010). When older individuals are given explicitly labeled false information, they tend to misremember the false information as true which influences judgments (Chen and Blanchard-Fields, 2000; Chen, 2002). This finding is not simply due to an impairment in source memory (Schacter et al., 1991), although source amnesia is theorized to result from a failure to “false tag” (Asp and Tranel, 2012) as increased suggestibility in older adults is correlated with source memory (Cohen and Faulkner, 1989). Rather, older people tend to misremember false information as true but show no problems misremembering true information as false (Chen and Blanchard-Fields, 2000; Chen, 2002). The unidirectional nature of the mistakes in belief is consistent with the FTT and suggests that the processes of belief and doubt are asymmetrical. Increased credulity during aging is associated with declines in prefrontal cortex functioning (Denburg et al., 2007); indeed, according to the frontal lobe aging hypothesis (West, 1996), there is a disproportionate decline in prefrontal cortex structural integrity and functionality in old age (Dempster, 1992; Pfefferbaum et al., 2005). In contrast to early development however, credulity in older persons cannot be explained by the lack of knowledge. If anything, older individuals tend to have increased knowledge and crystallized intelligence relative to younger individuals (Horn and Cattell, 1967). Here, age-related declines in ability of the prefrontal cortex to doubt provide a compelling rationale as to why highly knowledgeable and intelligent older people are often susceptible to deception and fraud (Figure 2).

### Neuroimaging evidence

Neuroimaging studies have confirmed that the prefrontal cortex is activated when doubt or disbelief must be employed. When stimulus conditions indicate an alteration of activated (and believed) representations, the prefrontal cortex should be critical toward “false tagging” these representations. Indeed, the prefrontal cortex is engaged when learned associations are contradicted (Fletcher et al., 2001), when evaluating data inconsistent with plausible theories (Fugelsang and Dunbar, 2004), when automatic lexical associations are violated by visual stimuli (Kerns et al., 2004), when rare events occur (Braver et al., 2001), when incongruous visual stimuli are presented (Michelon et al., 2003), when visual expectations are breached (Nobre et al., 1999), and when real-world beliefs are violated by visual illusions (Parris et al., 2009). Beyond the occurrence of unexpected events, activity in the prefrontal cortex is increased in situations of general **uncertainty** and decreased when situations are certain. In the FTT, uncertainty in outcomes is produced from multiple options activated in System 1 processing. Theoretically, as the number of activated responses and outcomes are increased, prefrontal “false tagging” should be increased to doubt various disadvantageous options. Task-related prefrontal activation decreases as the task becomes more familiar (Raichle et al., 1994; Grill-Spector et al., 2006; Race et al., 2009) and prefrontal activation increases as tasks become more difficult (D'Esposito et al., 1998; Nolde et al., 1998; Menon et al., 2000). In fact, prefrontal activation is quite sensitive to uncertainty changes as it tracks trial-by-trial changes in relative uncertainty during a temporal utility integration task (Badre et al., 2012).

As alluded to above, “false tagging” is intimately involved in choosing an advantageous response option during a decision making scenario (Asp and Tranel, 2012). Each choice is considered a belief representation (“if I choose X, then I get goal Y”) and prefrontal “false tags” negatively bias disadvantageous or inappropriate choices. Thus, selecting an advantageous option involves doubting, and thereby eliminating, the other disadvantageous options. Indeed, prefrontal activity is strongly correlated with increases in the number of response options (Marsh et al., 2007), the number of alternative outcomes (Elliott et al., 1999), and with subjective reports of choice difficulty (Arana et al., 2003). Further, when the availability of important probabilistic information regarding a response option is reduced (as in “ambiguous” uncertain choices), prefrontal cortex activity is also increased (Hsu et al., 2005; Huettel et al., 2006; Levy et al., 2010). Activation of the prefrontal cortex is predictive of both identifying disadvantageous outcomes and the evaluation of negative losses (Christakou et al., 2009) suggesting that the prefrontal cortex mediates behavioral shifting away from disadvantageous choices.

Taken together, the evidence indicates that the prefrontal cortex is critical in situations where expectations are violated, uncertainty is high, and ambiguity is increased. The FTT posits a general doubt toward task-relevant beliefs (i.e., associative activations of System 1) which may encapsulate these diverse findings. However, to this point, the prefrontal cortex has been considered a unitary region and the established subdivisions have been largely neglected. The next section examines the problem of functional localization in the prefrontal cortex and the FTT's view of functional subdivisions.

## Prefrontal functional localization

A common approach in science is the method of reductionism, where one attempts to understand complex phenomena by dividing them into simpler parts and studying their interactions. Investigations of the prefrontal cortex have proved no different as researchers have struggled to segregate regions in the prefrontal cortex based on functionality (e.g., Stuss et al., 2002; Van Veen and Carter, 2006). However, several problems unique to the prefrontal cortex cast doubt regarding this approach as they challenge the modular view of multiple independent functional units, where each subregion of the prefrontal cortex has distinct and non-overlapping functional processing.

First, several studies have indicated that a variety of tasks and phenomena can activate the same regions in the prefrontal cortex. Psychological demands as diverse as perception, response selection, task switching, problem solving, language, and episodic memory produce similar activations in the prefrontal cortex (Duncan and Owen, 2000). A recent review of neuroimaging studies examining dorsal anterior cingulate cortex (dACC) activation provides evidence that negative affect, pain, and cognitive control activate overlapping regions of the dACC (Shackman et al., 2011). There are several explanations that could account for fMRI activation overlap in a single prefrontal cortex subregion: (1) Different psychological tasks have the same functional requirement which is subserved by a single prefrontal cortex subregion that is unique in its functional properties. Here, the function of the subregion is distinct and non-overlapping with other prefrontal cortex subregions. Such an explanation is offered by Shackman et al. (2011) as an attempt to identify a single dACC function, by suggesting that the dACC uses punishment information to bias behavior in uncertain situations. (2) Different psychological tasks have the same functional requirement which is subserved by a prefrontal cortex subregion that is not unique in its functional properties. The function of a prefrontal cortex subregion is not unique and other subregions may provide the same function in cases of high demand. This hypothesis is favored by the FTT and may help account for the plasticity of the prefrontal cortex following damage (Stuss et al., 1987). (3) Different psychological tasks have different functional requirements which are subserved by a single prefrontal cortex subregion. In this model, distinct functions are either mediated by the same neural circuitry at different time points or differing functions are expressed at lower, single cell levels (e.g., Gilbert et al., 2010) which is often lost in large scale neuroimaging analyses. Moreover, other methodological limitations may contribute to common activations: (4) Different psychological tasks activate the same prefrontal subregion because of similar task impurities which produce systematic variance unrelated to the function of interest (see Miyake and Friedman, 2012). (5) Different psychological tasks activate the same prefrontal subregion because of common parallel input which has stronger correlation with the BOLD signal than spiking output (Logothetis et al., 2001; Wilson et al., 2010). Although these alternatives have not been systematically investigated, it is clear that a classification of distinct functions for the putative prefrontal cortex subregions based on neuroimaging results is problematic.

Using the methodology of single-unit neuronal recordings, prefrontal cortex neurons have been shown to be both (1) responsive to different tasks and conditions and (2) adaptable on the basis of current behavioral concerns (Asaad et al., 2000; Duncan and Miller, 2002). As task demands are increased, a greater amount of prefrontal neurons are recruited (Duncan and Miller, 2002). The increased prefrontal neuronal involvement may reflect either (1) additional distinct functions performed via the prefrontal cortex toward the task goal or (2) additional resource of the same function toward the task goal (Asp and Tranel, 2012). Indeed, this research suggests the putative subregions of the prefrontal cortex are adaptive and flexible to a variety of modalities and their associated specific tasks demands.

Second, different subregions of the prefrontal cortex can perform a single function. Lesions to the prefrontal cortex can produce reconfigurations of functionality are seen in contralesional homologous regions (Thulborn et al., 1999; Rosen et al., 2000) suggesting contralesional regional compensation (Stuss et al., 1987; Voytek et al., 2010). The functional reorganization may represent either (1) a new function subserved by the undamaged region or (2) a modification of an existing function subserved by the undamaged region toward a new modality. However, contralesional prefrontal compensation can occur on extremely short time scales as transcranial magnetic stimulation induced lesions produce compensation immediately after disruption (Lee and D'Esposito, 2012). This suggests that the compensatory prefrontal region does not need to “learn” a new function but can dynamically offer an existing function to task performance. Beyond the compensation from the contralesional hemisphere, multiple subregions within a hemisphere have been implicated in functional compensation of damage to the prefrontal cortex (e.g., dorsal lateral PFC, ventral lateral PFC, and anterior cinguate cortex compensations in working memory tasks; Hillary, 2008). However, it is also true that distinct lesions to the prefrontal cortex can produce selective deficits (e.g., Bechara et al., 1998; Asp and Tranel, 2012; Glascher et al., 2012; Tsuchida and Fellows, 2012). Traditionally, this has been used as evidence to suggest that a function is both unique to a prefrontal subregion and unadaptable by other regions following damage. However, this constructs a conflict between a plastic prefrontal cortex that can reconfigure following damage and a rigidly divided prefrontal cortex that cannot. The constraints and conditions that lead to one outcome over the other should be a primary aim of future research (Burgess and Robertson, 2002).

The FTT posits a **weak equipotentiality** principle for the prefrontal cortex where initial, low-demand processing for differing modalities is done at distinct local regions but as demand is increased or an orthogonal prefrontal process is engaged, additional prefrontal regions are recruited (see also Miller and Cohen, 2001). Regional modality inputs and outputs characterize functional distinctions; therefore, the inputs and outputs of different modalities to the prefrontal cortex determine what precise role (or executive function) “false tagging” is contributing to. For instance, dorsal prefrontal regions often “false tag” attentional representations to produce attentional focus and ventral prefrontal regions often “false tag” cognitive representations to doubt cognitions. However, in the FTT this segregation is not exclusive. In cases of high demand or lesions (with modality access to other prefrontal regions), “false tagging” from other regions of the prefrontal cortex can be supplied. Thus, the “normative” segregation of functions is driven by which region receives the initial information from a particular modality (e.g., Seeley et al., 2007). We theorize that the plasticity of prefrontal cortex function following damage is primarily dependent on the access of different modalities to undamaged prefrontal regions. Structural white matter analyses in prefrontal cortex patients may yield evidence toward this hypothesis as the prefrontal cortex is both highly (1) interconnected (Barbas and Pandya, 1989, 1991) and (2) connected to parietal and temporal association cortices (Petrides and Pandya, 2002). In this model the prefrontal cortex performs the singular function of “false tagging,” a resource for which different modalities can compete. If the FTT is accurate, a reductionist fractionation of the prefrontal cortex may be illusory.

## Prefrontal cortex control

The **executive system** is a theorized cognitive process that controls and manages other psychological functions. The functions of the executive system have been defined as four related processes: planning, decision-making, judgment, and self-perception (Tranel et al., 1994). While the executive system has been useful as a psychological construct, its appeal as a quantifiable cognitive/behavior capacity is suspect (Tranel et al., 1994), and theoretically it suffers from the **homunculus fallacy**, where the very psychological properties under investigation are explained by an internal device (Donald, 1991; Allport, 1993). There is a strong consensus that the prefrontal cortex plays an important role in biasing attention and behavior in executive functions (Norman and Shallice, 1986; Hazy et al., 2007)—however, without a specific mechanism of *how* the prefrontal cortex mediated executive “decides” to bias attention and behaviors, the concept will remain homuncular. In addition, a comprehensive account of a circumscribed role for the prefrontal cortex in all theorized executive functions must be postulated to avoid ragbag effects, whereby any process not well understood is categorized under the executive system (Baddeley, 1996). The FTT offers a single function for the prefrontal cortex, which plays a key, specific role in the broader executive functions. The three principles derived from System 1 (perpetual associative activations, activated representations as beliefs, and regional segregation) and the “false tagging” function of the prefrontal cortex may offer a new view of executive control that is not as dependent on homuncular concepts.

Activated mental representations in the FTT are potent and dynamic as they automatically activate a cascade of other coherent cognitions, behaviors, facial expressions, autonomic perturbations, and emotions (Gilbert, 1993). This view is in contrast to the traditional computational perspective where mental representations are impotent and static; and an additional controller must perform complex operations with the static representations (e.g., Baddeley, 1996). The FTT posits that during a situation requiring an executive function (such as a decision making scenario) a series of task-relevant cognitions are elicited from System 1 associative activations. These activations can be the specific task cognitions (if I choose X, I get Y), but they also can be other relevant task cognitions (e.g., probabilistic information: I have a 20% chance of getting Y). When two or more mutually incompatible representations are activated, a negative somatic state is created (Festinger, 1957; Damasio, 1994; Asp and Tranel, 2012). The established neural networks associated with the cognitions produce cognitive inconsistencies that are indexed on the level of emotions and prediction errors (Asp and Tranel, 2012). The negative weighting of each option is determined by the activated associative characteristics toward each specific task cognition (e.g., probabilistic information, affective characteristics, ambiguity information, and goal attribute information). This negative somatic state is affixed to the “untrue” or disadvantageous option via the prefrontal cortex, and “untrue” or disadvantageous “false tagging” can be done multiple times until no other incompatible representations are found or activated. The “false tags” are biasing signals toward mental representations in parietal and temporal association cortices. Thus, the prefrontal mediated “false tags” produce an inhibition of activated neural networks for a “disadvantageous” mental representation. The “false tags” decreased the likelihood of a behavioral response for that option by its inhibition. “False tags” work on mental representations outside of the prefrontal cortex. In the FTT, every potential response option or incompatible belief does not need to be represented. In fact, evidence suggests that individuals often fail to consider alternative options even in decision-making scenarios with several obvious options; instead, people will behave and believe according to the initial automatic activations of System 1 (Kahneman, 2011). However, when other options are represented, they are automatically subjected to an evaluation and potential “false tagging” (for a description of the automatic and unpredictable results of this process see Gilbert, 1993). The choice of representations is strongly biased by System 1's activation pattern and strength which can greatly influence executive functions such as decision-making without prefrontal cortex mediation.

This model suggests that the prefrontal cortex is a blind and dumb selector of neural network “states” that are primed to behave. The selection is produced by active suppression of the disadvantageous or “untrue” representations that are activated. Associative activations of mental representation that identify discrepancies, discordant information, and negative characteristics produce the “false tags” of the prefrontal cortex. The precise description of how this process occurs is a matter for future research but the FTT suggests that monoamine-driven prediction errors play a central role in the production of “false tags” (Asp and Tranel, 2012). This model does not eliminate homuncular critiques completely from executive control, but it does offer a parsimonious account of prefrontal functioning and highlights remaining questions for the neural operations underlying executive functioning.

## Heuristics and biases in judgment and decision-making

For the last 40 years, psychological research has seen an explosion of studies examining heuristics, or mental short-cuts, during judgment and decision-making (Griffin et al., 2001; Gilovich and Griffin, 2002). This line of research has shown that individuals will engage in systematic heuristics during judgments rather than “rationally” combining subjective probability and utility to arrive at an expected utility, which would provide the optimal outcome (Griffin et al., 2001). Instead, people reliably make “intuitive” judgments that are not rational but are based on a series of principles such as representativeness, availability, and anchoring-and-adjustment (Tversky and Kahneman, 1974). Moreover, it is not that individuals preferentially choose to make judgments with heuristics rather than a “rational” assessment; rather, heuristical thinking is natural; it is the fundamental process by which people arrive at judgments and decisions. Indeed, heuristical thinking is widespread even under ideal conditions of high motivation, high ability, and high effort (Griffin et al., 2001) However, even if heuristical thinking is indeed a property of the mind (rather than a choice of it), this still begs the question of *why* individuals use a system prone to such biases and errors. The FTT proposes that heuristical thinking and its biases stem from the properties of neural systems and how they interact. While the “heuristics and biases” program has invaded theoretical development in many fields (Gilovich and Griffin, 2002), it has been conspicuously absent in neuroanatomical models of executive functioning (a noteworthy exception is the explanation of the affective heuristic by the Somatic Marker Hypothesis, Damasio, 1994). Here, we offer a neural-based hypothesis for a heuristical process to examine the potential benefits of the FTT in this domain.

**Figure 3:**
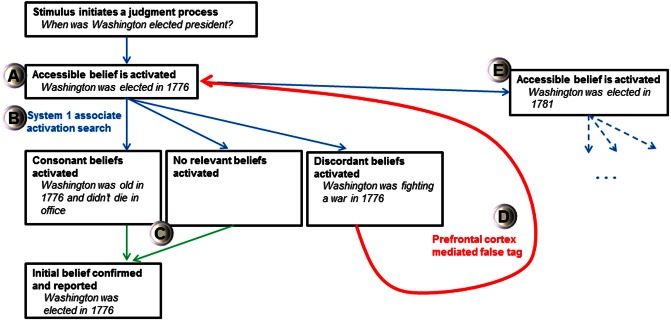
**Schematic of False Tagging Theory's anchor-and-adjustment process.** Bold text represents the stage and italicized text is an example of a self-generated anchor process (Epley and Gilovich, 2001). Blue arrows represent System 1 association activation processes, green arrows represent behavioral output processes, and the red arrow represents false tagging mediated by the prefrontal cortex. See text for discussion of the various stages and **(A–E)** designations. Only activation of discordant beliefs can initiate a false tag and produce a new belief representation with a larger anchor-estimate gap.

The FTT's view of all activated cognitions as beliefs and a doubting “false tag” initiated by a least two discrepant beliefs may help explain how and why intuitive judgments produce biases. For instance, the FTT may explain why people use an anchor-and-adjustment judgment process, where accessible cognitions strongly influence estimates of unknown quantities (Tversky and Kahneman, 1974; Epley and Gilovich, 2001). In the FTT, anchor effects arise because initial cognitions from the easily accessible value are believed (considered true). First, a *belief* anchored by accessible knowledge is activated after a judgment stimulus is apprehended (Figure 3A). Then, a search is conducted by associated activations of System 1, which will find one of three primary results relating to the initial belief: consonant beliefs, no relevant beliefs, or discordant beliefs (Figure 3B). If consonant beliefs or no relevant beliefs are found, then the initial belief is confirmed and reported as accurate (Figure 3C). The initial belief can only be falsified if discordant beliefs are found during a search process. If a discordant belief is activated, the prefrontal cortex can affix a false tag to the initial belief (Figure 3D). When individuals have directional certainty knowledge (Simmons et al., 2010), a new value is searched for (which is associatively coherent with the old value), represented, and believed (Figure 3E). As before, a search process is initiated for this belief which produces consonant, discordant, or no relevant beliefs. This process will continue until no discordant belief is found and no “false tag” can be attached to the belief under current scrutiny. Here, merely considering an anchor increases the plausibility of values around it (Simmons et al., 2010) because the initial anchor is truly believed and the nature of the falsification process is dependent on an effortful and uncertain search process that is not guaranteed to find discordant information. The FTT suggests that the prefrontal cortex is critical for “doubting” initial anchors which are automatically believed. If there is limited time, little motivation, an inadequate search, no critical discordant information to be found, or a dysfunctional prefrontal “false tagging” process, then estimations should have large anchor effects (prefrontal patients can produce bizarre estimations suggesting failed adjustment from a self-generated anchor, see Shallice and Evans, 1978). Thus, if this hypothesis is proved correct, a model of potent and believed cognitions with prefrontal “false tags” has the potential to alleviate homunculus critiques of executive functioning and may explain many biases prevalent in heuristical psychology.

## Conclusion

Our demonstration that prefrontal patients are generally credulous to external information is the first empirical evidence for the FTT (Asp et al., 2012a). The results have implications of considerable breadth, from societal issues such as aging and marketing ethics to theoretical issues in neuroscience such as models of executive function and how heuristics operate in the brain. Our findings suggest a strong asymmetry in the way we arrive at beliefs and disbeliefs. Beliefs are inherent in the associative process of thought, while disbelief is retroactive, difficult, and governed by a distinct neural process (Gilbert, 1991; Asp and Tranel, 2012). The evidence suggests that we are perpetually-moving belief machines with feeble doubting brakes (Kahneman, 2011). Initial beliefs often have a stronger influence than later discrepant beliefs (e.g., Asch, 1946), which may explain biases such as anchoring effects, halo effects (Kahneman, 2011), the perseverance effect (Ross et al., 1975), and the correspondence bias (Gilbert, 1991). Indeed, while the perspective of the prefrontal cortex as a “doubter” may offer benefits to neuroanatomical models of executive function, it may also suggest a neuroanatomical rationale for why we often give the “benefit of the doubt” itself: secondary doubt is simply weaker and less reliable than initial belief.

### Conflict of interest statement

The authors declare that the research was conducted in the absence of any commercial or financial relationships that could be construed as a potential conflict of interest.

## Key Concept

False Tagging Theory: A neuroanatomical model of the belief and doubt processes. The False Tagging Theory proposes that a unitary function of the prefrontal cortex is to “false tag” for various modalities. Damage to the prefrontal cortex creates a “doubt deficit” and a tendency toward belief and credulity.
False tagging: The reductive essence of the psychological concept of doubt in cognition and the singular neuroanatomical function of the prefrontal cortex in the False Tagging Theory. The prefrontal cortex affixes false tags to perceptual and cognitive representations to negatively bias distractions, beliefs, judgments, and decisions.
System 1: The fast, automatic, and associative process in dual process models of psychological functioning. System 1 functioning leads to many of the biases in judgment and decision making in heuristical psychology.
Doubt deficit: A failure to produce normative levels of doubt for a particular item of information. In the False Tagging Theory, doubt is a secondary process that inhibits information that is already believed. A doubt deficit will produce increases in belief to novel information that is primarily represented.
Uncertainty: A knowledge state of having limited knowledge to exactly describe a knowable quantity, a future outcome, or multiple outcomes. An uncertain outcome where probability can be confidently judged is termed risky uncertainty. An uncertain outcome where probabilistic or important information is missing is termed ambiguous uncertainty.
Weak equipotentiality: A neurological principle where low-demand processing is done at distinct local neural regions but high-demand processing engages additional neural regions which may interfere with orthogonal processes.
Executive system: A theoretical entity which conducts a host of related cognitive processes including planning, decision-making, judgment, and self-perception. The executive acts to control and manage other “lower level” cognitive processes.
Homunculus fallacy: A theoretical argument used to denounce accounts of psychological processes which are hollow or redundant, because they attribute an internal, unspecified device to the psychological process under description.
